# Investigating the Mechanisms of 15-PGDH Inhibitor SW033291 in Improving Type 2 Diabetes Mellitus: Insights from Metabolomics and Transcriptomics

**DOI:** 10.3390/metabo14090509

**Published:** 2024-09-20

**Authors:** Yuanfeng Huang, Mingjie Liang, Yiwen Liao, Zirui Ji, Wanfen Lin, Xiangjin Pu, Lexun Wang, Weixuan Wang

**Affiliations:** 1 Traditional Chinese Medicine Research Institute, Guangdong Pharmaceutical University, No. 280, Waihuan East Road, University Town, Guangzhou 510006, China; 2112148137@stu.gdpu.edu.cn (Y.H.); 2112048023@stu.gdpu.edu.cn (M.L.); 2112242088@stu.gdpu.edu.cn (Y.L.); 2112242063@stu.gdpu.edu.cn (Z.J.); 2112348029@stu.gdpu.edu.cn (W.L.); 2112348040@stu.gdpu.edu.cn (X.P.); wanglexun@gdpu.edu.cn (L.W.); 2 Guangdong Provincial Research Center of Integration of Traditional Chinese Medicine and Western Medicine in Metabolic Diseases, Guangzhou 510006, China; 3 Guangdong Nephrotic Drug Engineering Technology Research Center, Guangdong Consun Pharmaceutical Group, Institute of Consun Co. for Chinese Medicine in Kidney Diseases, Guangzhou 510700, China

**Keywords:** type 2 diabetes mellitus, SW033291, 15-hydroxyprostaglandin dehydrogenase, metabolomics, transcriptomics

## Abstract

This study focused on exploring the effects of SW033291, an inhibitor of 15-hydroxyprostaglandin dehydrogenase, on type 2 diabetes mellitus (T2DM) mice from a comprehensive perspective. Studies have demonstrated that SW033291 benefits tissue repair, organ function, and muscle mass in elderly mice. Our recent investigation initially reported the beneficial effect of SW033291 on T2DM progression. Herein, we used a T2DM mouse model induced by a high-fat diet and streptozotocin injection. Then, serum and liver metabolomics, as well as liver transcriptomic analyses, were performed to provide a systematic perspective of the SW033291-ameliorated T2DM. The results indicate SW033291 improved T2DM by regulating steroid hormone biosynthesis and linoleic/arachidonic acid metabolism. Furthermore, integrated transcriptomic and metabolomic analyses suggested that key genes and metabolites such as *Cyp2c55*, *Cyp3a11*, *Cyp21a1*, *Myc*, *Gstm1*, *Gstm3*, 9,10-dihydroxyoctadecenoic acid, 11-dehydrocorticosterone, and 12,13-dihydroxy-9Z-octadecenoic acid played crucial roles in these pathways. qPCR analysis validated the significant decreases in the hepatic gene expressions of *Cyp2c55*, *Cyp3a11*, *Myc*, *Gstm1*, and *Gstm3* in the T2DM mice, which were reversed following SW033291 treatment. Meanwhile, the elevated mRNA level of *Cyp21a1* in T2DM mice was decreased after SW033291 administration. Taken together, our findings suggest that SW033291 has promising potential in alleviating T2DM and could be a novel therapeutic candidate.

## 1. Introduction

Diabetes mellitus is a common disease worldwide that results in significant rates of impairment and death. Recent epidemiological investigations indicated that there are about 537 million individuals, aged 20–79, affected by diabetes mellitus, making up roughly 10.5% of the global populace [1]. Unfortunately, this number is anticipated to escalate rapidly, with an estimated 783 million adult diabetics projected by 2045, resulting in a considerable public financial burden [1]. In 2021, the costs associated with diabetes mellitus reached an astonishing USD 966 billion in healthcare spending [2,3]. Type 2 diabetes mellitus (T2DM) accounts for over 90% of all diabetes mellitus cases. Nevertheless, the current conventional strategies used to treat T2DM frequently have some adverse effects, underlining the pressing need to develop innovative therapeutic interventions [4].

SW033291 is a potent and high-affinity inhibitor of 15-hydroxyprostaglandin dehydrogenase (15-PGDH), an enzyme responsible for the conversion of prostaglandin E_2_ (PGE_2_) into its less biologically active metabolite, 15-keto-PGE_2_ [5]. Characterized as a pale yellow to yellowish-green powder, SW033291 has a molecular formula of C_21_H_20_N_2_OS_3_, and its structure is shown in Supplementary Figure S1. This compound exhibits a melting point above 99 °C and possesses a low Ki value of 0.1 nM, which is indicative of its strong binding affinity. Furthermore, SW033291 demonstrates high solubility in DMSO, a property that is particularly advantageous for its application in biological assays and drug delivery systems.

Structural studies have revealed that the residues Ser138, Tyr151, and Gln148 on 15-PGDH, which are crucial for the enzyme’s function, are directly involved in interactions with SW033291 [6]. Moreover, the binding affinity is further enhanced by hydrophobic interactions involving additional residues including Leu139, Ala140, Val145, Gly184, Phe185, Ile190, Leu191, Ser193, Ile194, Ile210, Ile214, Tyr217, and Thr246 [6]. Notably, the butyl side chain of SW033291, which includes the sulfoxide moiety, engages in key binding interactions with 15-PGDH that are akin to those observed in the C15-OH and C16-C20 alkyl chains of PGE_2_ [6]. These interaction studies underscore the molecular basis for SW033291’s inhibitory effect on 15-PGDH, suggesting the great potential of SW033291 in treating prostaglandin pathway-related diseases.

PGE_2_ is a crucial lipid signaling molecule that promotes tissue repair and regeneration as well as improving various pathological processes [5,7,8,9,10]. Studies have demonstrated that PGE_2_ can improve insulin resistance and inflammation caused by fatty acids, as well as decrease the activity of enzymes involved in lipogenesis to prevent hepatic steatosis [7,8,9]. Moreover, PGE_2_ inhibits the synthesis of tumor necrosis factor-α (TNF-α), while decreased PGE_2_ synthesis results in elevated TNF-α levels, which enhances IL-1β generation and causes hepatocyte apoptosis [10]. A lack of hepatic PGE_2_ leads to lipid accumulation and liver inflammation in non-alcoholic fatty liver disease (NAFLD) [11]. Furthermore, the overexpression of cyclooxygenase-2, the rate-limiting enzyme in PGE_2_ biosynthesis, in diabetic mice has been shown to ameliorate the pro-apoptotic state of the liver [12]. The aforementioned studies indicate a potential protective role of PGE_2_ in the context of T2DM. However, PGE_2_ can also exhibit pro-inflammatory effects in certain conditions, highlighting the dual nature of its role in inflammation. Historically, interventions involving prostaglandins have largely focused on their inhibition, with clinically established inhibitors such as indomethacin, ibuprofen, and paracetamol being widely utilized for their anti-inflammatory and analgesic effects through the suppression of prostaglandin synthesis [13]. However, our research introduces SW033291 as a novel and intriguing exception to this trend, attributed to its effect of elevating PGE_2_ levels. Various recent studies demonstrated that SW033291 promoted tissue regeneration, prevented kidney and liver injury, and improved muscle function by elevating PGE_2_ levels, indicating that SW033291 can protect against numerous metabolic diseases [5,14,15,16,17]. Together, these results indicate that SW033291 can ameliorate metabolic disorders, such as T2DM, making it a compelling subject for further investigation.

Our recent investigation initially showed that SW033291 administration exerted protective roles on T2DM mice by improving their aberrant glucose metabolism [18]. Due to the significant potential of SW033291 as a therapeutic option for managing T2DM, we conducted a comprehensive assessment of its protective properties against T2DM and explored the mechanisms involved through a combined analysis of metabolomics and transcriptomics. Our research offered a systematic understanding of the salutary impact of SW033291 on T2DM, potentially enhancing the use of SW033291 in preventing and treating T2DM.

## 2. Materials and Methods

### 2.1. Animals and Drugs

All animals were housed in an SPF-grade environment at the Experimental Animal Center of Guangdong Pharmaceutical University. Food and water were provided ad libitum to the animals throughout the experimental process, while the temperature was maintained at 20–26 °C. Male C57BL/6 mice, aged seven weeks, were procured from Zhuhai BesTest Bio-technology Co., Ltd. (Zhuhai, China). The SW033291 reagent was sourced from MedChemExpress (HY-16968, Monmouth Junction, NJ, USA). The Ethics Review Committee of Guangdong Pharmaceutical University approved this animal experiment (Approval No. gdpulacspf2017556). This animal study was conducted following the guidelines outlined in the National Research Council’s Manual for the Treatment and Handling of Laboratory Animals.

### 2.2. Experimental Protocol for Animals

After acclimatization for 1 week, all mice were randomly divided into three different groups (*n* = 6 each). The SW033291-treated group received twice-daily intraperitoneal injections of a single dose of 5 mg/kg SW033291 (dissolved in 85% dextrose-5 water, 10% ethanol, and 5% Cremophor EL) for 10 weeks, 2 weeks before high-fat diet (HFD; HF60, Dyets, Bethlehem, PA, USA) feeding [18]. In parallel, the control and model groups were injected with vehicle controls and received a standard diet. Mice in both the SW033291-treated group and the model group were fed an HFD for 2 weeks. Subsequently, they received intraperitoneal injections of streptozotocin (S0130, Sigma-Aldrich, St Louis, MO, USA; dissolved in citrate buffer) at a dosage of 30 mg/kg/day for 3 consecutive days to induce T2DM. Throughout the experiment, these mice continued to be fed the HFD. In parallel, the control group received injections of citrate buffer for 3 consecutive days and continued to be fed a standard dirt. One week after streptozotocin injection, fasting blood glucose levels were measured. In the model group, mice with fasting blood glucose levels exceeding 11.1 mmol/L were considered to be T2DM mice and were utilized for subsequent experiments [19]. The animal experiment process is shown in Supplementary Figure S1.

### 2.3. Sample Collection

After 12 h of fasting, all of the mice were anesthetized and blood samples were collected by means of retro-orbital bleeding. Then, all mice were humanely euthanized through cervical dislocation. The blood samples were subjected to centrifugation at 4 °C for 15 min at 3500 rpm. After that, the supernatant was moved to a fresh tube and kept at −80 °C for subsequent experimentation. The liver tissue samples were rapidly obtained and promptly frozen at −80 °C.

### 2.4. Metabolomics Investigation Utilizing Liquid Chromatography-Tandem Mass Spectrometry (LC-MS/MS)

#### 2.4.1. Sample Preparation and Extraction

The serum samples (50 μL) were thawed on ice and briefly vortexed before being mixed with 300 μL of an extraction solution made of 20% acetonitrile in methanol with internal standards. Internal standards, including L-2-chlorophenylalanine, [2H3]-L-carnitine HCl, and 4-fluoro-L-α-phenylglycine, are added to the extraction solvent for quality control and to monitor the stability of the experimental process. After vortexing the mixture for 3 min, it was centrifuged at 4 °C for 10 min at 12,000 rpm. Afterward, 200 μL of the supernatant was moved to a fresh tube and stored at −20 °C for 30 min. After being centrifuged at 12,000 rpm for 3 min at 4 °C, 180 μL of the supernatant was moved to the appropriate injection vial for further examination.

Regarding the analysis of liver metabolomics, after liver samples were thawed, 20 mg of liver tissue was homogenized and centrifuged for 30 s at 3000 rpm at 4 °C. Following centrifugation, 400 μL of a 70% methanol solution with internal standards, including L-2-chlorophenylalanine, [2H3]-L-carnitine HCl, and 4-fluoro-L-α-phenylglycine, was added and shaken for 5 min, chilled on ice for 15 min, and then centrifuged at 4 °C at 12,000 rpm for 10 min. After that, 300 μL of the resulting supernatant was moved to a fresh tube and stored for 30 min at −20 °C. After centrifuging at 4 °C at 12,000 rpm for 3 min, 200 μL of the resulting supernatant was transferred to the appropriate injection vial for further experimentation.

#### 2.4.2. Instrumentation and Data Analysis

The samples obtained were examined using a LC-ESI-MS/MS system (UPLC, ExionLC AD, SCIEX, Framingham, MA, USA; MS, QTRAP 6500, SCIEX), utilizing a ACQUITY UPLC HSS T3 C18 chromatographic column (1.8 µm, 2.1 mm × 100 mm) (Waters, Milford, MA, USA). The column temperature was set to 40 °C, with a flow rate of 0.4 mL/min. The injection volume was 2 μL. The solvent system was water (0.1% formic acid)/acetonitrile (0.1% formic acid). The gradient program was 95%:5% *v*/*v* at 0 min, 10%:90% *v*/*v* at 11.0 min, 10%:90% *v*/*v* at 12.0 min, 95%:5% *v*/*v* at 12.1 min, and 95%:5% *v*/*v* at 14.0 min.

Linear ion trap (LIT) and triple quadrupole (QQQ) scans were acquired using a QTRAP^®^ LC-MS/MS system, a triple quadrupole-linear ion trap mass spectrometer, fitted with an ESI Turbo Ion-Spray interface. Positive and negative ionization modes were both used and managed by Analyst 1.6.3 software (SCIEX). The ESI source settings were as follows: the source temperature was set at 500 °C, with ion spray voltages of 5500 V for positive mode and −4500 V for negative mode. The ion source gases, GSI and GSII, along with the curtain gas, were set at 55, 60, and 25.0 psi, respectively. The collision gas for collision-activated dissociation (CAD) was set high. The mass spectrometer was calibrated and tuned using polypropylene glycol solutions at concentrations of 10 and 100 μmol/L in QQQ and LIT modes, respectively. For each period, a specific set of multiple reaction monitoring (MRM) transitions was monitored according to the eluted metabolites within this method.

To ensure the reliability of the detection and filtering findings, a quality control procedure was implemented. Quality control samples were prepared by pooling sample extracts, which allows for the assessment of repeatability across analyses conducted under the same processing conditions. Within the instrumental analysis sequence, a quality control sample was inserted after every 8–10 analytical samples to continuously monitor the analytical process’s repeatability. The repeatability of metabolite extraction and detection was evaluated by conducting an overlay analysis of the total ion chromatogram obtained from the mass spectrometric analysis of different quality control samples. The coefficients of variation (CV) value reflect the degree of data dispersion. The detected substance information was filtered based on the CV of the quality control samples.

The mass spectrometry data were analyzed by Analyst 1.6.3 software, and qualitative and quantitative analyses of the metabolites were performed on the samples. Utilizing a self-compiled targeted metabolite database MWDB (metware database, Metware Biotechnology Co., Ltd., Wuhan, China), qualitative analysis was performed based on the retention time, characteristic ion pair information, and secondary mass spectrometric data of the analytes. For quantitative analyses of the metabolites, the characteristic ions of each substance were selected by a triple quadrupole, and the signal intensity of the characteristic ions was obtained in the detector. The raw mass spectrometry files of the samples were opened with MultiQuant software (version 3.0.3) (SCIEX), and the integration and correction of the chromatographic peaks were performed. The peak area of each chromatographic peak represents the relative content of the corresponding substance. Finally, all the integrated data of the chromatographic peak areas were exported and saved.

Metabolites with significant differences were chosen based on *p* < 0.05 and VIP > 1. Additionally, the compound names of the metabolites were uploaded to MetaboAnalyst 5.0 for the enrichment analysis of metabolic pathways. Principal component analysis (PCA), Orthogonal partial least squares−discriminant analysis (OPLS-DA), and volcano map analysis were performed using R (“ggplot2” package) and MetaboAnalyst 5.0. The metabolomic data generated in this study have been deposited to the MetaboLights (https://www.ebi.ac.uk/metabolights/) database (Accessed on 6 September 2024) under the accession number MTBLS11026.

### 2.5. Transcriptomic Analysis

#### 2.5.1. Total RNA Extraction

Total RNA was isolated from the liver tissues using Trizol (Invitrogen, Carlsbad, CA, USA) following the manufacturer’s protocol. Approximately 60 mg of liver tissue was pulverized into powder under liquid nitrogen in a 2 mL tube, homogenized for 2 min, and then left to sit for 5 min. The homogenate was centrifuged at 12,000× *g* for 5 min at 4 °C, and the resulting supernatant was transferred to a fresh tube containing 0.3 mL of chloroform/isoamyl alcohol (24:1). This mixture was vortexed for 15 s and centrifuged at 12,000× *g* for 10 min at 4 °C. The supernatant, rich in RNA, was carefully transferred to a new tube and mixed with an equal volume of isopropyl alcohol. After centrifugation at 13,600 rpm for 20 min at 4 °C, the supernatant was discarded, and the RNA pellet was washed with 1 mL of 75% ethanol, followed by centrifugation at 4 °C for 3 min at 13,600 rpm to remove residual ethanol, and the pellet was air-dried in a biosafety cabinet for 5–10 min. The RNA was then resuspended in 25–100 μL of DEPC-treated water. RNA quality and concentration were assessed using a NanoDrop spectrophotometer and an Agilent 2100 bioanalyzer (Thermo Fisher Scientific, Waltham, MA, USA).

#### 2.5.2. mRNA Library Construction, Sequencing, and Data Analysis

Magnetic beads with oligo (dT) were utilized for mRNA enrichment. The isolated mRNA was fragmented using a breaking buffer, and reverse transcription was carried out to synthesize double-stranded cDNA. The synthesized double-stranded DNA was then end-repaired and phosphorylated to create a 3′-overhanging “A” sticky end, which was ligated with a bubble-shaped adaptor with a 3′-overhang “T”. Subsequently, using specific primers, the ligation products were amplified via PCR, followed by purification using Ampure XP Beads (1000006383, MGI, Shenzhen, China) and resuspension in EB buffer. Quality assessment of the PCR products was conducted using the Agilent 2100 bioanalyzer. The PCR products were first denatured to single-stranded DNA, then circularized into a single-stranded circular DNA library using a bridging primer. This single-stranded circular DNA was prepared as the definitive library. This library was then amplified using phi29 to create DNA nanoballs (DNBs), each containing over 300 molecules. These DNBs were loaded into a nanoarray and paired-end 150 base reads were generated on the MGIseq2000 platform (BGI, Shenzhen, China).

To ensure the precision of the data, we utilized SOAPnuke software (version 1.5.6) to filter out reads that had adaptors, an unknown nucleotide N content exceeding 5%, and reads with a low-quality base ratio (≤15) surpassing 20%. After obtaining the clean data, HISAT2 was utilized for mapping the clean reads to the reference genome, and Ericscript (version 0.5.5) and rMATS (version 3.2.5) were utilized for the detection of fusion genes and differential splicing genes, respectively. Bowtie2 was employed for aligning the filtered reads to the gene set. Gene expression quantification was performed using RSEM software (version 1.3.1).

DESeq2 was utilized for analyzing differentially expressed genes. Genes that exhibit significant differential expression were selected based on a Q value ≤ 0.05 and log2FC > 1 or log2FC < −1.

To analyze genes that were differentially expressed, we conducted functional classification and enrichment analysis. Gene Ontology (GO) and Kyoto Encyclopedia of Genes and Genomes (KEGG) were utilized to analyze differentially expressed genes through Phyper enrichment analysis. Functions with a Q value ≤ 0.05 were deemed significantly enriched. In addition, a network of protein–protein interactions (PPI) was created to explore the interactions between differentially expressed genes. The STRING website (https://string-db.org/) (version 11.5) was utilized to create the PPI network and identify disease-related genes. This work has been registered under NCBI BioProject PRJNA1157743, and the raw RNA sequencing data can be accessed in the Sequence Read Archive (SRA) database with the accession numbers SAMN43534804, SAMN43534805, SAMN43534806, SAMN43534807, SAMN43534808, SAMN43534809, SAMN43534810, SAMN43534811, SAMN43534812, SAMN43534813, SAMN43534814, and SAMN43534815.

### 2.6. Integrated Analyses of Transcriptomics and Metabolomics

We conducted integrated pathway analyses of the differentially changed genes and metabolites utilizing MetaboAnalyst 5.0. The relationship between metabolites and genes, along with the mechanism of action of SW033291 on T2DM mice, was revealed by MetaboAnalyst 5.0. We used the R (“corrplot” package) to draw metabolite and gene correlation heatmaps, which are more intuitive for presenting the correlation between metabolites and genes.

### 2.7. Real-Time Quantitative PCR (qPCR)

Total RNA was isolated from hepatic tissues utilizing the Trizol reagent, obtained from Dongsheng (Guangzhou, China). After extracting the RNA, it was converted into cDNA through reverse transcription using the instructions provided in the reverse transcription kit (RR047A, Takara, Japan). The LightCycler 480 system (Roche, Basel, Switzerland) was used for the qPCR experiment. The levels of expression of the specific genes were measured utilizing the 2^−ΔΔCT^ approach. Ruiboxingke Biotechnology Co., Ltd. (Beijing, China) produced the primers. The following are the primer sequences: *Myc*: forward 5′-CCTTCTCTCCTTCCTCGGAC-3′, reverse 5′-TTCTTGCTCTTCTTCAGAGTCG-3′; *Cyp3a11*: forward 5′-GTGCTCCTAGCAATCAGCTT-3′, reverse 5′-GTCGAATTTCCATAAACCCTTGTA-3′; *Gstm1*: forward 5′-CTGTTACAACCCTGACTTTGTGG-3′, reverse 5′-TGGCTGTCACCACCTTTAGAC-3′; *Gstm3*: forward 5′-AGCACAACCTGTGTGGAGAG-3′, reverse 5′-TCGGGACTGCAGCAGACTAT-3′; *Cyp2c55*: forward 5′- TGACCCTGGCCATTTTCTGG-3′, reverse 5′-ACGCACATTCGCTTTCCTAT-3′; *Cyp21a1*: forward 5′-CTTGGGGATGCAAGATGTGGT-3′, reverse 5′-GCAAAGTCCACCCACTTTTGG-3′; *Actb*: forward 5′-GCTCCGGCATGTGCAAAG-3′, reverse 5′-TTCCCACCATCACACCCTGG-3′. *Actb* served as an internal control.

### 2.8. Statistical Analysis

The results were analyzed employing the GraphPad Prism software (version 9.0) (La Jolla, CA, USA). The data were analyzed using one-way ANOVA followed by Tukey’s test. We deemed statistical significance to be *p* < 0.05.

## 3. Results

### 3.1. Analysis of Serum Metabolomics

A model of T2DM in mice was established through the administration of an HFD, coupled with the injection of streptozotocin. In our previous study [18], the T2DM model mice exhibited significantly elevated levels of fasting blood glucose, low-density lipoprotein cholesterol, triglycerides, and total cholesterol, when compared to the control group, while treatment with SW033291 led to a significant decrease in serum glucose and lipid levels. Moreover, SW033291 improved glucose tolerance and reduced insulin resistance in mice with T2DM [18]. Prior research indicates that SW033291 has a beneficial impact on T2DM mice and could be a promising treatment approach for inhibiting T2DM advancement. However, the underlying mechanisms of SW033291’s effect on T2DM deserve further investigation.

**Figure 1:**
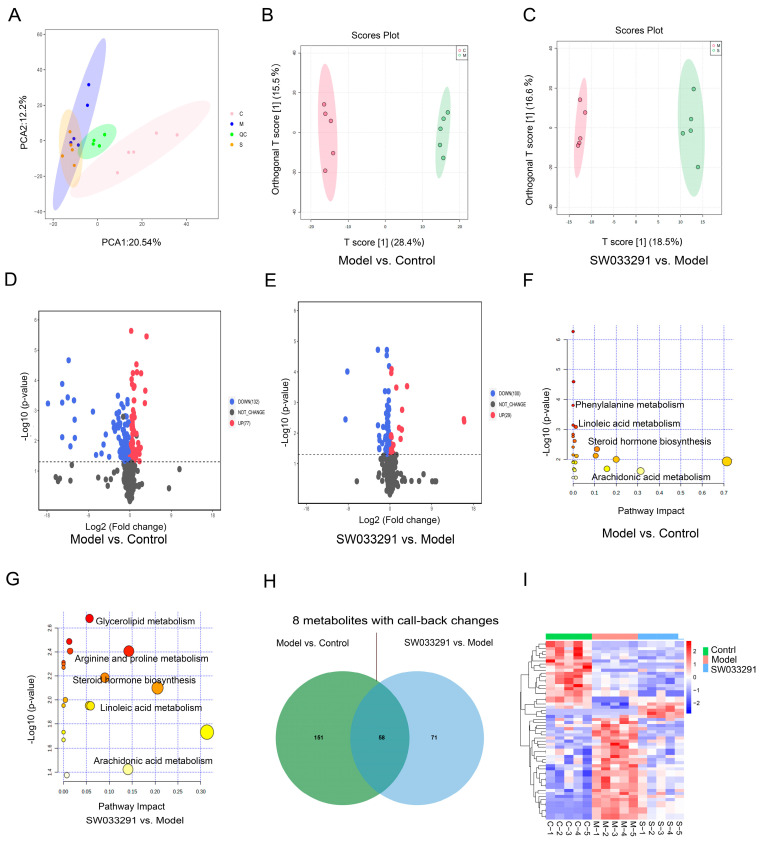
Comprehensive serum metabolomic analysis in control, model, and SW033291−treated mice, revealing distinct metabolic profiles. (**A**) The principal component analysis (PCA) score plot illustrates the metabolic variance among control, model, and SW033291−treated groups, highlighting the separability of metabolic profiles. Orthogonal partial least squares−discriminant analysis (OPLS−DA) plots comparing (**B**) “Model vs. Control” and (**C**) “SW033291 vs. Model” groups, showcasing the group discrimination. (**D**,**E**) Volcano maps for “Model vs. Control” and “SW033291 vs. Model” comparisons, depicting the fold change and statistical significance of individual serum metabolites, where up−regulated and down−regulated metabolites are indicated by red and blue, respectively. Metabolites above the dashed line in the volcano plot indicate a *p*-value of less than 0.05. (**F**,**G**) KEGG pathway enrichment analysis of significantly changed serum metabolites in “Model vs. Control” and “SW033291 vs. Model” comparisons, identifying dysregulated metabolic pathways. The deeper the color of the circle, and the closer it is to red, the smaller the *p*-value. (**H**) Venn diagram representing the number of unique and shared serum metabolites with significant changes in “Model vs. Control” and “SW033291 vs. Model” comparisons. (**I**) Clustered heatmap of the 58 overlapping and differentially changed serum metabolites, with color intensity reflecting the magnitude of up−regulation (red) or down−regulation (blue).

Serum metabolomics analysis was conducted to detect alterations in metabolites in the control, model, and SW033291-treated mice. The overall differences in the serum metabolome and the level of variability within the three groups were depicted using PCA (Figure 1A). OPLS-DA revealed a clear distinction between the model and control groups, as well as between the SW033291-treated and model groups (Figure 1B,C). In this study, differentially changed metabolites were defined as those with VIP > 1 and *p* < 0.05. The volcano plots revealed 77 up-regulated and 132 down-regulated metabolites in the model group when compared with the control group (Figure 1D). SW033291-treated mice had 29 up-regulated and 100 down-regulated metabolites in comparison to the model group (Figure 1E). KEGG enrichment analysis was conducted using MetaboAnalyst 5.0, using the differentially altered metabolites in “Model vs. Control” and “SW033291 vs. Model” comparisons. Metabolites that were differentially changed between the model and control groups were mainly associated with phenylalanine metabolism, steroid hormone biosynthesis, linoleic acid metabolism, and arachidonic acid metabolism (Figure 1F). The metabolites that were differentially changed in response to SW033291 treatment compared to the model group were primarily annotated and enriched in glycerolipid metabolism, arginine and proline metabolism, linoleic acid metabolism, steroid hormone biosynthesis, and arachidonic acid metabolism (Figure 1G). There were shared metabolic pathways in these comparisons, such as arachidonic acid metabolism, steroid hormone biosynthesis, and linoleic acid metabolism, as depicted in Figure 1F,G. In the comparisons between “Model vs. Control” and “SW033291 vs. Model”, the Venn diagram indicates 58 overlapping and differentially changed metabolites, such as 11-dehydrocorticosterone, 12,13-epoxyoctadecenoic acid (12,13-EpOME), and 9,10-epoxyoctadecenoic acid (9,10-EpOME) (Figure 1H and Supplementary Table S1). Next, we conducted a cluster heatmap analysis, and the levels of overlapping and differentially changed metabolites among these three groups are shown in Figure 1I. The heatmap showed that eight of the overlapping and differentially changed metabolites have a marked trend of “high–low–high” or “low–high–low”, such as 11-dehydrocorticosterone, 9,10-EpOME, 12,13-EpOME, rhapontigenin, Glu-Thr, and phytosphingosine, which were deemed as key serum metabolites in SW033291-ameliorated T2DM. It is important to mention that the levels of 9,10-EpOME, 12,13-EpOME, and 11-dehydrocorticosterone were lower in the model group compared to the control group, and these alterations were reversed following the administration of SW033291.

### 3.2. Analysis of Liver Metabolomics

**Figure 2:**
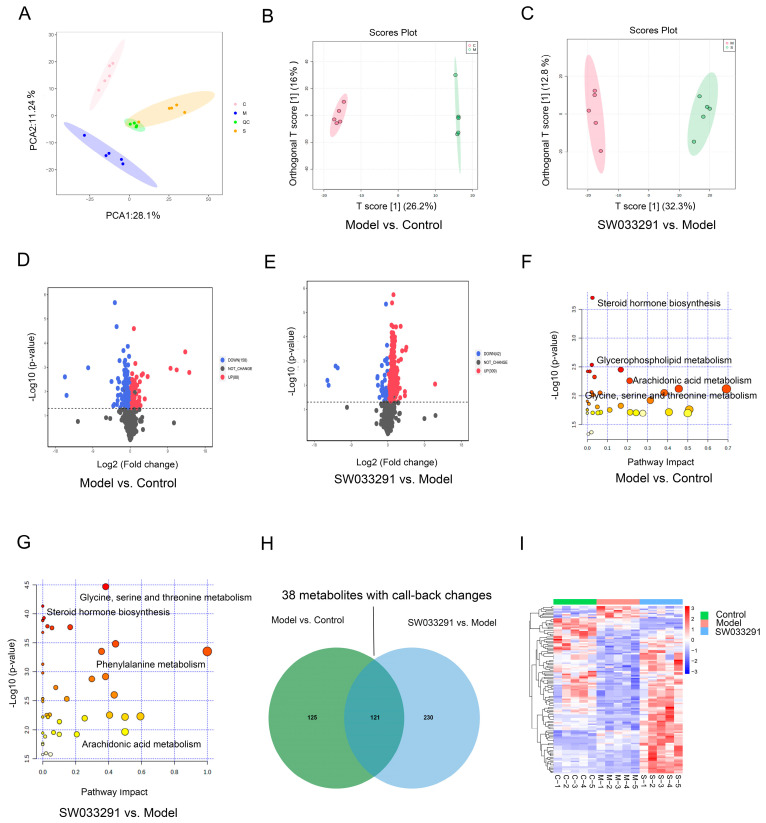
Hepatic metabolomic profiling in control, model, and SW033291−treated mice, uncovering treatment−specific metabolic alterations. (**A**) PCA score plot reveals the distinct metabolic fingerprints among the three groups. OPLS−DA plots comparing (**B**) “Model vs. Control” and (**C**) “SW033291 vs. Model” groups, presenting the group discrimination. (**D**,**E**) Volcano maps for “Model vs. Control” and “SW033291 vs. Model” comparisons, depicting the fold change and statistical significance of hepatic metabolites, where up−regulated and down−regulated metabolites are shown in red and blue, respectively. Metabolites above the dashed line in the volcano plot indicate a *p*-value of less than 0.05. (**F**,**G**) KEGG pathway enrichment analysis of significantly changed hepatic metabolites in the “Model vs. Control” and “SW033291 vs. Model” comparisons, providing insights into the metabolic pathways affected by SW033291 treatment. The deeper the color of the circle, and the closer it is to red, the smaller the *p*-value. (**H**) Venn diagram illustrating the number of unique and overlapping hepatic metabolites with significant changes in “Model vs. Control” and “SW033291 vs. Model” comparisons. (**I**) Clustered heatmap of 121 overlapping and differentially changed hepatic metabolites, with color intensity reflecting the magnitude of up−regulation (red) or down−regulation (blue).

Due to the predominant role of the liver in the pathogenesis of T2DM [20], we hypothesized that SW033291 ameliorated T2DM by modulating the hepatic metabolic profile. Liver metabolomic analysis was performed to identify differentially changed metabolites among control, model, and SW033291-treated mice. PCA and OPLS-DA demonstrated distinct metabolic patterns among the three groups (Figure 2A–C). Differentially changed metabolites were screened using *p* < 0.05 and VIP > 1. According to the volcano plot, 88 metabolites had increased levels and 158 metabolites had decreased levels in the model group compared with the control group (Figure 2D). In contrast, the SW033291-treated group displayed 309 up-regulated metabolites and 42 down-regulated metabolites compared with the model group (Figure 2E). These differentially changed metabolites underwent KEGG enrichment analysis using MetaboAnalyst 5.0. The results indicate that the differentially changed metabolites between the control and the model group were mainly annotated and enriched in metabolic pathways such as arachidonic acid metabolism, glycerophospholipid metabolism, steroid hormone biosynthesis, and glycine, serine, and threonine metabolism (Figure 2F). In contrast, the differentially changed metabolites between the model and the SW033291-treated group were mainly annotated and enriched in metabolic pathways such as phenylalanine metabolism, glycine, serine, and threonine metabolism, arachidonic acid metabolism, and steroid hormone biosynthesis (Figure 2G). Some metabolic pathways overlapped with these comparison groups, such as glycine, serine, and threonine metabolism, steroid hormone biosynthesis, and arachidonic acid metabolism, as shown in Figure 2F,G. In the comparisons between “Model vs. Control” and “SW033291 vs. Model”, the Venn diagram displays 121 metabolites that were differentially changed and overlapping (Figure 2H and Supplementary Table S2). Then, we performed a cluster heatmap analysis, which showed that 38 of the overlapping and differentially changed metabolites have a marked trend of “high–low–high” or “low–high–low”, such as 9,10-dihydroxyoctadecenoic acid (9,10-DiHOME), 11-dehydrocorticosterone, 12,13-dihydroxy-9Z-octadecenoic acid (12,13-DiHOME), (±)-alpha-CMBHC, carnitine C8:0, and N-acetylaspartate, which were deemed as key hepatic metabolites in SW033291-ameliorated T2DM (Figure 2I). Additionally, it is worth noting that SW033291 increased the levels of 12,13-DiHOME, 9,10-DiHOME, and 11-dehydrocorticosterone in the livers of the model group mice.

### 3.3. Analysis of Liver Transcriptomics

**Figure 3:**
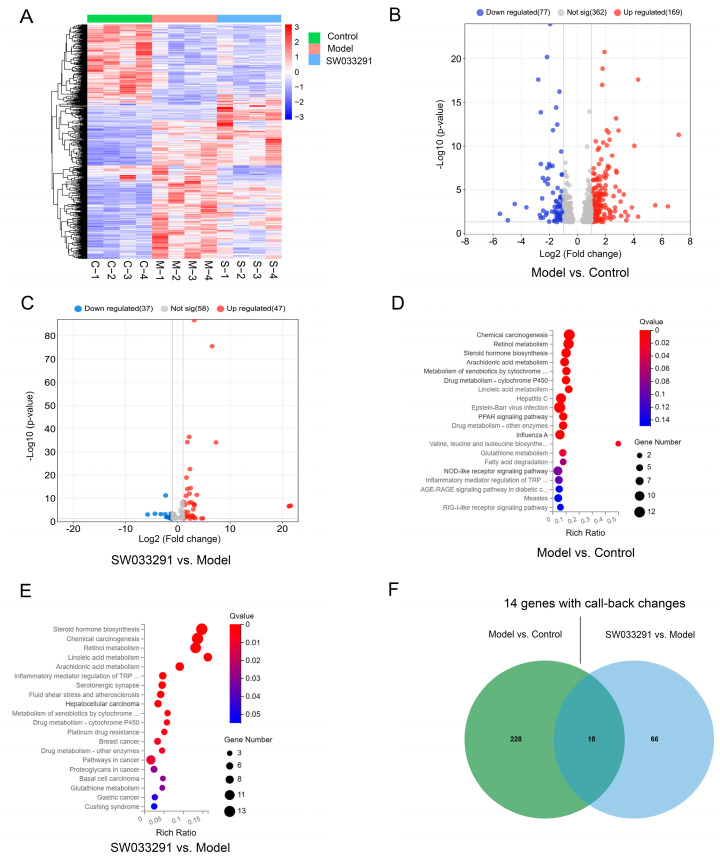
Comparative hepatic transcriptomics profiling in Control, Model, and SW033291−treated mice. (**A**) Cluster heatmap analysis depicting the expression patterns of hepatic genes across the three groups, with color gradients indicating the magnitude of up−regulation (red) or down−regulation (blue). (**B**,**C**) Volcano maps for “Model vs. Control” and “SW033291 vs. Model” comparisons, visualizing the statistical significance of and fold change in gene expression. (**D**,**E**) KEGG pathway enrichment analysis for the “Model vs. Control” and “SW033291 vs. Model” comparisons, pinpointing the biological processes and pathways influenced by the treatment. (**F**) Venn diagram displaying the number of distinct and shared significantly altered hepatic genes.

To gain a deeper insight into the molecular pathways impacted by SW033291 in the livers of mice with T2DM, liver transcriptome profiling was conducted on the control, model, and SW033291-treated mice. A total of 17,006 genes were detected, and after filtering out adapter sequences and substandard reads, genes specifically expressed among the three groups were visualized in a heatmap, revealing distinct differences in liver gene expression (Figure 3A). The volcano plot shows that 246 genes were differentially expressed between the control and model mice, including 169 up-regulated and 77 down-regulated genes (Figure 3B). Following SW033291 treatment, 84 genes were differentially expressed between the model and SW033291-treated mice, with 47 genes being up-regulated and 37 genes being down-regulated (Figure 3C). Following that, an analysis of KEGG pathway enrichment was conducted, revealing that the genes with significantly altered expression were primarily associated with steroid hormone biosynthesis, retinol metabolism, linoleic acid metabolism, and arachidonic acid metabolism pathways in the comparisons between the model and control groups (Figure 3D). After SW033291 treatment, the above-mentioned pathways were also affected, indicating that SW033291 may ameliorate T2DM by affecting these pathways (Figure 3E).

**Figure 4:**
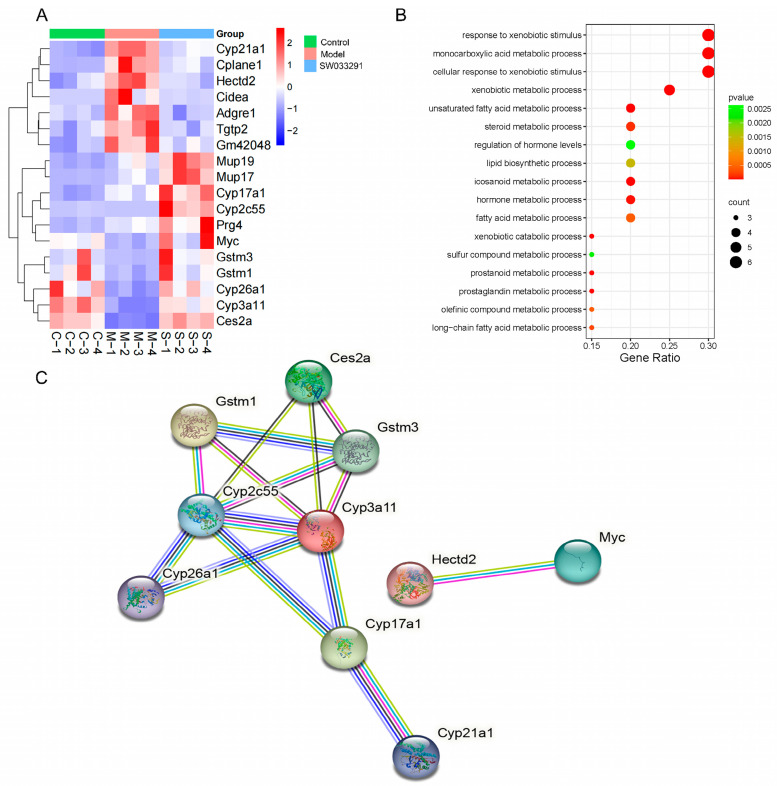
Detailed transcriptomic analysis of liver tissue identifying key genes and pathways in control, model, and SW033291−treated mice. (**A**) Cluster heatmap analysis highlighting the expression of 18 key genes that are differentially expressed across three groups. Gene expression changes are represented by red (up−regulated) and blue (down−regulated) colors. (**B**) Gene Ontology analysis enriches biological processes associated with the 18 overlapping and differentially expressed genes, offering insights into the affected cellular processes and molecular mechanisms. (**C**) Protein−protein interaction network analysis based on the 18 overlapping and significantly altered genes, revealing potential functional associations and regulatory networks. Disconnected nodes are not shown in the network.

The Venn diagram depicts 18 overlapping and differentially expressed genes between the “Model vs. Control” and “SW033291 vs. Model” comparisons (Figure 3F and Supplementary Table S3). Cluster heatmap analysis showed that 14 overlapping and differentially expressed genes have a significant trend of “high–low–high” or “low–high–low”, such as steroid 21-hydroxylase (*Cyp21a1*), ciliogenesis and planar polarity effector 1 (*Cplane1*), glutathione S-transferase Mu 3 (*Gstm3*), glutathione S-transferase Mu 1 (*Gstm1*), pyrethroid hydrolase Ces2a (*Ces2a*), and cytochrome P450 3A11 (*Cyp3a11*) (Figure 4A). To understand the related cellular processes of these overlapping and differentially expressed genes, we conducted GO enrichment analysis, revealing that these genes were primarily engaged in processes such as steroid metabolic, unsaturated fatty acid metabolic, and lipid biosynthetic processes (Figure 4B). Furthermore, using PPI network analysis, we found that *Gstm1*, *Gstm3*, *Cyp3a11*, cytochrome P450 2C55 (*Cyp2c55*), and *Cyp21a1* had high connectivity with other genes, underscoring their significance in SW033291-ameliorated T2DM (Figure 4C).

### 3.4. Integrated Analyses of Metabolomics and Transcriptomics Unveiled the Mechanisms Underlying SW033291’s Effects in the Treatment of T2DM

**Figure 5:**
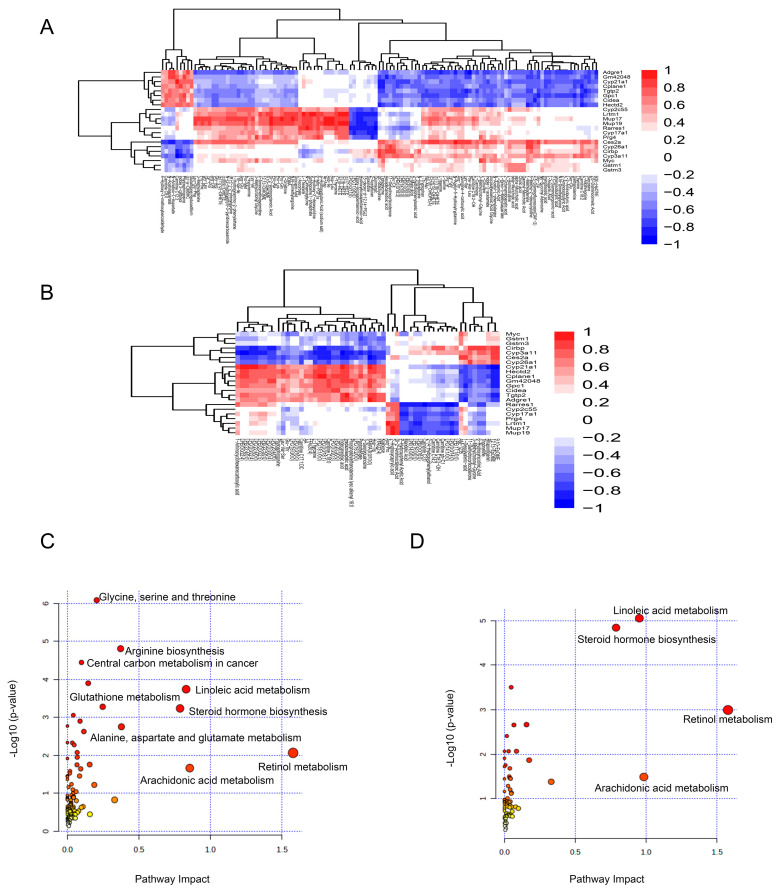
Integrative analysis of metabolomics and transcriptomics reveals complex interactions between gene expression and metabolite profiles. (**A**) Heat map depicting the associations between differentially changed genes and metabolites in the combined analyses of liver transcriptomics and liver metabolomics. (**B**) Combined analyses of liver transcriptomics and serum metabolomics. In these heat maps, correlations are color−coded: −1 < r < 0 indicates a negative correlation, visualized in blue; 0 < r < 1 indicates a positive correlation, visualized in red; and r = 0 indicates no correlation, visualized in white. The intensity of the color reflects the strength of the correlation, providing a quantitative measure of the association between gene expression and metabolite levels. Joint-pathway analysis of (**C**) liver transcriptomics and liver metabolomics and (**D**) liver transcriptomics and serum metabolomics identifying convergent pathways affected by SW033291 treatment.

To investigate the correlation between differentially expressed genes and differentially changed metabolites, we calculated the Pearson correlation coefficients and depict them in Figure 5A,B. Our combined analysis of liver transcriptomics and liver metabolomics revealed a positive correlation between *Cyp3a11* and 11-dehydrocorticosterone, whereas *Cyp21a1* displayed a negative correlation with 11-dehydrocorticosterone (Figure 5A). In the comprehensive analysis of liver transcriptomics and serum metabolomics, *Cyp3a11* remains positively correlated with 11-dehydrocorticosterone, and *Cyp21a1* is still negatively correlated with 11-dehydrocorticosterone (Figure 5B). The above findings indicate a significant correlation between the differentially changed genes and metabolites identified in our study.

Furthermore, MetaboAnalyst5.0 was used to perform integrated transcriptomic and metabolomic analyses. The enriched pathways for integrated analyses of liver transcriptomics and liver metabolomics including the central carbon metabolism, glutathione metabolism, steroid hormone biosynthesis, linoleic acid metabolism, and arachidonic acid metabolism (Figure 5C). 9,10-DiHOM, 12,13-DiHOME, *Cyp2c55*, and *Cyp3a11* were simultaneously enriched in the linoleic acid metabolism pathway; L-glutamic acid, L-ornithine, *Gstm1*, and *Gstm3* were simultaneously enriched in the glutathione metabolism pathway; and 11-dehydrocorticosterone, *Cyp21a1*, *Cyp3a11*, and steroid 17-alpha-hydroxylase/17, 20 lyase (*Cyp17a1*) were simultaneously enriched in the pathway of steroid hormone biosynthesis (Figure 5C and Supplementary Table S4). Based on the combined analyses of liver transcriptomics and serum metabolomics, the enriched pathways included steroid hormone biosynthesis, linoleic acid metabolism, arachidonic acid metabolism, and retinol metabolism (Figure 5D). Arachidonic acid, 13 (R)-HODE, *Cyp2c55*, and *Cyp3a11* were simultaneously enriched in the linoleic acid metabolism pathway; 11-dehydrocorticosterone and genes such as *Cyp21a1*, *Cyp3a11*, and *Cyp2c55* were simultaneously enriched in the steroid biosynthesis pathway; and arachidonic acid and *Cyp2c55* were simultaneously enriched in the arachidonic acid metabolism pathway (Figure 5D and Supplementary Table S5).

Therefore, our comprehensive analyses of metabolomics and transcriptomics shed light on the relationship between differentially changed genes and metabolites and identify key metabolic pathways related to SW033291-ameliorated T2DM, which may offer potential targets for further exploration.

### 3.5. Verification of Significantly Altered Genes by qPCR

**Figure 6:**
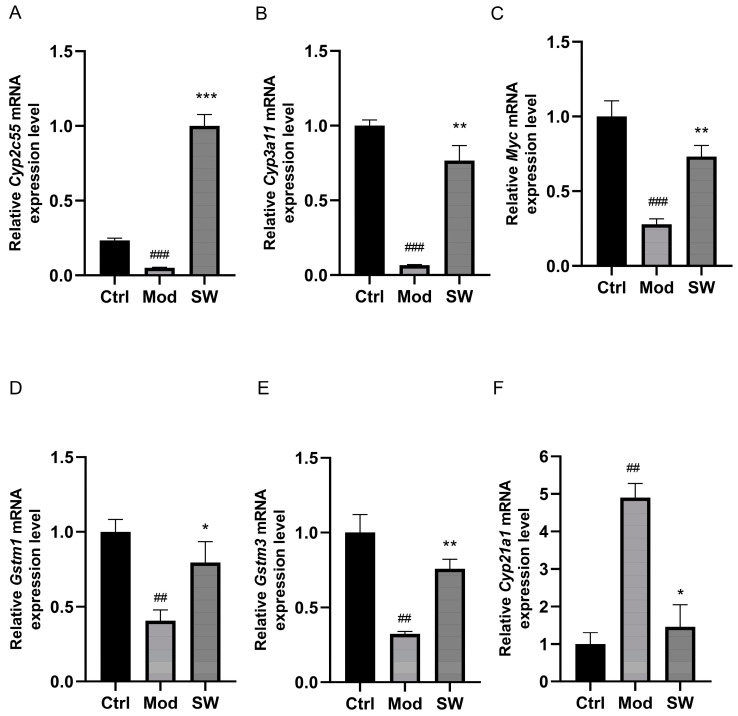
Quantitative mRNA expression analysis of select genes in liver tissue from control, model, and SW033291−treated mice. The livers from the control (Ctrl), model (Mod), and SW033291−treated (SW) group were analyzed for hepatic mRNA levels of (**A**–**F**) *Cyp2c55*, *Cyp3a11*, *Myc*, *Gstm1*, *Gstm3*, and *Cyp21a1*, respectively. n = 6. Data are presented as mean ± SEM. ^##^ *p* < 0.01, ^###^ *p* < 0.001 vs. the Ctrl group; * *p* < 0.05, ** *p* < 0.01, *** *p* < 0.001 vs. the Mod group.

Significant decreases in the gene expression of *Cyp2c55*, *Cyp3a11*, Myc proto-oncogene protein (*Myc*), *Gstm1*, and *Gstm3* were observed in the livers of T2DM mice compared to the control group (Figure 6A–E). Following treatment with SW033291, there was a notable increase in the expression of these genes (Figure 6A–E), which was aligned with the transcriptomic analysis. On the contrary, the mRNA levels of *Cyp21a1* were elevated in the livers of T2DM mice but decreased following SW033291 treatment (Figure 6F).

## 4. Discussion

T2DM is a global public health challenge, characterized by substantial morbidity and mortality rates, highlighting the imperative for innovative therapeutic strategies. PGE_2_ is a lipid signaling molecule that plays a role in a range of physiological and pathological processes and benefits multiple metabolic disorders, including T2DM and NAFLD [11,12]. SW033291 is a compound that inhibits 15-PGDH, an enzyme responsible for breaking down PGE_2_ [5]. Recent findings indicate that SW033291 enhanced tissue regeneration and repair by significantly increasing PGE_2_ levels [5,14,15,16]. Despite the evidence that SW033291 could be a beneficial treatment approach for enhancing tissue regeneration and repair, further studies on its effects and mechanisms on T2DM are necessary. The current study conducted a systematic investigation into the mechanisms of SW033291 in treating T2DM using a combination of metabolomics and transcriptomics. Our findings reveal that SW033291 alleviated T2DM by influencing pathways related to linoleic acid metabolism, steroid hormone biosynthesis, and arachidonic acid metabolism. Key genes and metabolites involved included *Cyp2c55*, *Cyp3a11*, *Cyp21a1*, *Myc*, *Gstm1*, *Gstm3*, 11-dehydrocorticosterone, 9,10-DiHOME, and 12,13-DiHOME.

Myc is crucial in various cellular activities like cell growth, proliferation, and differentiation [21]. Research indicates that the appropriate induction of Myc expression in humans or rodents can increase β-cell replication without the risk of cell death or impaired insulin secretion, indicating the possible benefits of Myc in treating T2DM [22]. More importantly, a study showed that hepatic overexpression of Myc prevented insulin resistance and obesity [23]. Our combined analyses of hepatic transcriptomics with hepatic or serum metabolomics revealed a reduction in Myc expression levels in the livers of T2DM mice, which was reversed after treatment with SW033291, indicating that the beneficial effects of SW033291 on T2DM mice may be achieved by up-regulating Myc expression.

Cyp3a11 and Cyp2c55 belong to the cytochrome P450 enzyme group, which is widely distributed in the liver and other tissues and is essential for metabolizing drugs as well as endogenous substances within the human body [24]. *Cyp3a11* plays critical roles in the metabolic processing of a wide range of endogenous and exogenous drugs, as well as various lipid compounds [24]. Research has demonstrated that during lipopolysaccharide (LPS)-induced liver injury, LPS significantly inhibits the mRNA level of *Cyp3a11* in mice, indicating the positive role of Cyp3a11 in protecting against liver damage [25]. Furthermore, studies conducted on male rats with bile duct ligation-induced cholestasis have shown a decrease in the gene expression of *Cyp2c55* [26], suggesting a positive role of Cyp2c55 in metabolic diseases. In our combined analyses of hepatic transcriptomics with hepatic or serum metabolomics, we observed down-regulated gene expression levels of *Cyp3a11* and *Cyp2c55* in T2DM mice, which were enhanced following SW033291 treatment. These findings suggest that SW033291 could alleviate T2DM by increasing the levels of *Cyp2c55* and *Cyp3a11* and provide protection against liver injury.

GSTM1 belongs to the glutathione S-transferase family and mainly acts as a detoxification enzyme [27]. Several studies have indicated that the absence of the Gstm1 gene might elevate the risk of developing T2DM [28] and increase the susceptibility to kidney failure and heart failure [29]. GSTM3 is a phase II detoxification enzyme that maintains redox homeostasis [30]. GSTM3 is widely recognized for its detoxifying properties and its ability to eliminate reactive oxygen species [30]. In diabetic and insulin-resistant mice, the gene expression levels of *Gstm3* are reduced [31]. Meanwhile, an increase in *Gstm3* expression might alleviate liver fibrosis, and Gstm3 could serve as a potential radiotherapy target for patients with hepatocellular carcinoma [32,33]. The integration of transcriptomics and metabolomics in our study showed reduced hepatic levels of *Gstm1* and *Gstm3* in T2DM mice, but their levels increased after treatment with SW033291, indicating that SW033291 may up-regulate the expression of *Gstm1* and *Gstm3* to improve oxidative stress, enhance antioxidant activity, and thereby ameliorate T2DM.

CYP21A1 is a member of the cytochrome P450 enzyme family, which is predominantly involved in the biosynthesis of steroid hormones [34]. Steroid hormones are a class of lipids that include sex hormones and adrenal cortex hormones [35]. Adrenal cortex hormone can be divided into glucocorticoids and mineralocorticoids, with glucocorticoids including cortisol, cortisone, and corticosterone, and mineralocorticoids including aldosterone [35]. The expression and activity changes displayed by CYP21A1 contribute to the synthesis and secretion of steroid hormones [36,37]. Research has demonstrated a notable rise in *Cyp21a1* levels in obese and T2DM mice, highlighting the importance of steroid hormones in obesity and T2DM [38]. 11-dehydrocorticosterone can activate the inactivated glucocorticoids [39]. Research has shown that glucocorticoids can promote hyperglycemia, dyslipidemia, insulin resistance, hepatic steatosis, and central obesity progression [40], and dysregulation of glucocorticoid signaling may contribute to the pathogenesis of metabolic syndrome [41], indicating the key role of 11-dehydrocorticosterone in T2DM progression. Our current research utilized transcriptomic and metabolomic analyses to identify *Cyp21a1* and 11-dehydrocorticosterone as crucial genes and metabolites. Analysis of hepatic transcriptomics showed the up-regulation of *Cyp21a1* levels in the model mice, which were down-regulated following the administration of SW033291. Metabolomic analysis of the liver or serum revealed reduced levels of 11-dehydrocorticosterone in T2DM mice, which were elevated after treatment with SW033291. These findings indicate that SW033291 may reduce the production of glucocorticoids by lowering *Cyp21a1* expression, thereby improving T2DM.

12,13-DiHOME and 9,10-DiHOME are derived from linoleic acid [42], which is an essential polyunsaturated fatty acid in the human body [43]. Cytochrome P450 enzymes are involved in the metabolism of linoleic acid, leading to the production of 12,13-EpOME and 9,10-EpOME, which are later converted to 12,13-DiHOME and 9,10-DiHOME, respectively [42]. Research indicated that targeting 12,13-DiHOME and 9,10-DiHOME could be beneficial in treating obesity and its sequelae [44]. Research suggests that 9,10-DiHOME has beneficial effects on systemic metabolism [44]. A recent study recognized brown adipose tissue as a secretory organ that primarily secretes 12,13-DiHOME [45]. Reports indicate that targeting 12,13-DiHOME may help to decrease the chances of developing obesity, T2DM, and cardiovascular diseases. The injection of 12,13-DiHOME can lower serum triglyceride levels [46] and counteract the detrimental effects of an HFD on cardiac function and remodeling [47]. Our combined analyses of hepatic transcriptomics and hepatic metabolomics reveal that 12,13-DiHOME and 9,10-DiHOME are crucial hepatic metabolites that were reduced in the model group and restored following the administration of SW033291. This suggests that SW033291 may alleviate the occurrence and development of T2DM by reducing triglyceride levels, modulating mitochondrial metabolism, and inhibiting inflammation.

As a novel inhibitor, SW033291 shows significant potential in treating diabetes, and our group first reported that SW033291 ameliorated T2DM [18]. Other studies have highlighted the reparative capabilities of SW033291 in various injury models, including liver resection surgeries [16], intestinal damage [16], and bone marrow failure [48]. Moreover, combined therapy with SW033291 and PGE_2_ has improved the success rate of cervical ripening in mice [49]. Reduced PGE_2_ levels, associated with systemic anaphylaxis in mice, can be mitigated by inhibiting 15-PGDH [50]. Furthermore, increased 15-PGDH activity in aged mice leads to PGE_2_ depletion and muscle loss [17]. These findings suggest that SW033291 could be translated into clinical applications, particularly in promoting tissue repair and regeneration in patients undergoing bone marrow transplants, liver resections, or other surgical interventions. Its ability to regulate PGE_2_ levels also implies the potential to prevent or alleviate conditions related to PGE_2_ depletion, such as systemic anaphylaxis or age-related muscle loss. Future clinical studies are necessary to validate these benefits and to assess the efficacy of SW033291 in human treatment. However, this compound’s potential hepatotoxicity, as predicted in a study using pkCSM [51], warrants careful consideration. Although we did not observe any side effects during the 10-week treatment period, it is necessary to pay attention to the side effects of SW033291 over a longer duration in follow-up studies. This will help to determine its therapeutic potential and safety in clinical settings.

There are both strengths and limitations in the present study. The current study is the first comprehensive examination of the mechanistic impacts of SW033291 on T2DM by combining liver transcriptomics and liver/serum metabolomics, which is important for the future development of SW033291 or other 15-PGDH inhibitors for clinical use. However, this study also possesses certain limitations as it solely focuses on the liver and the serum, whereas the progression of T2DM involves multiple organs and tissues. Additional research can be conducted on the pancreas or other tissues that respond to insulin, including skeletal muscle and adipose tissue. Moreover, in this study, we selected male mice. There are two primary reasons for this: firstly, male mice are known to be more susceptible to streptozotocin-induced diabetes than female mice and rats [52,53,54]. This susceptibility can be quite pronounced, with female mice showing a minimal response to streptozotocin, while males develop severe hyperglycemia when administered the same dose [55]. Secondly, the hormonal fluctuations associated with the estrous cycle in female mice could potentially influence the experimental outcomes [56]. In contrast, male mice exhibit relatively stable hormone levels, which reduces variability in the experiments. However, as only male mice were used in this study, without considering the potential influence of female mice on steroid hormone metabolism-related pathways, future research should delve into the outcomes when female mice are employed. Despite the possibility that gender differences between male and female mice may impact the experimental results, generally speaking, the synthesis of steroid hormones, whether in males or females, originates from cholesterol [57], and certain enzymes (such as CYP) involved in this process are present in both sexes [58]. Therefore, we believe that there are some commonalities between male and female mice. Despite the limitations noted, our study still provides valuable insights into the molecular mechanisms of SW033291 in ameliorating T2DM, which can serve as a foundation for future research.

## 5. Conclusions

In conclusion, our research elucidates the therapeutic mechanisms of the 15-PGDH inhibitor SW033291 in T2DM. Through integrating liver transcriptomics, liver metabolomics, and serum metabolomics, our research demonstrates that the anti-T2DM capability of SW033291 may be associated with disturbances in the biosynthesis of steroid hormone as well as the metabolism of arachidonic acid and linoleic acid. Furthermore, key genes and metabolites including *Cyp2c55*, *Cyp3a11*, *Cyp21a1*, *Myc*, *Gstm1*, *Gstm3*, 11-dehydrocorticosterone, 12,13-DiHOME, and 9,10-DiHOME played crucial roles in the above-mentioned signaling pathways. Therefore, our work provides a novel perspective on the mechanisms of the 15-PGDH inhibitor SW033291 in treating T2DM, suggesting that SW033291 has the potential to be developed as a therapeutic for T2DM. Furthermore, it can be inferred that targeting the above-mentioned pathways and related genes or metabolites could also be developed as potential treatments for T2DM.

## Data Availability

The original contributions presented in this study are included in this article/the Supplementary Materials; further inquiries can be directed to the corresponding author/s.

## Supplementary Materials

The following supporting information can be downloaded at: https://www.mdpi.com/article/10.3390/metabo14090509/s1, Supplementary Table S1: The overlapping differentially changed metabolites between “Model vs. Control” and “SW033291 vs. Model” comparisons in serum metabolomics; Supplementary Table S2: The overlapping differentially changed metabolites between “Model vs. Control” and “SW033291 vs. Model” comparisons in liver metabolomics; Supplementary Table S3: The overlapping differentially expressed genes between “Model vs. Control” and “SW033291 vs. Model” comparisons in liver transcriptomics; Supplementary Table S4: The integrated analysis of liver transcriptomics and liver metabolomics; Supplementary Table S5: The integrated analysis of liver transcriptomics and serum metabolomics; Supplementary Figure S1: Animal experiment process.

## Abbreviations

9,10-DiHOME, 9,10-dihydroxyoctadecenoic acid; 9,10-EpOME, 9,10-epoxyoctadecenoic acid; 12,13-DiHOME, 12,13-dihydroxy-9Z-octadecenoic acid; 12,13-EpOME, 12,13-epoxyoctadecenoic acid; 15-PGDH, 15-hydroxyprostaglandin dehydrogenase; *Cyp2c55*, cytochrome P450 2C55; *Cyp3a11*, cytochrome P450 3A11; *Cyp21a1*, steroid 21-hydroxylase; *Cyp17a1*, steroid 17-alpha-hydroxylase/17,20 lyase; Cplane1, ciliogenesis and planar polarity effector 1; *Ces2a*, pyrethroid hydrolase Ces2a; *Gstm1*, glutathione S-transferase Mu 1; *Gstm3*, glutathione S-transferase Mu 3; HFD, high-fat diet; LPS, lipopolysaccharide; *Myc*, Myc proto-oncogene protein; NAFLD, non-alcoholic fatty liver disease; PGE_2_, prostaglandin E_2_; T2DM, type 2 diabetes mellitus; TNF-α, tumor necrosis factor-α.
